# Temperature Resolution Improvement in Raman-Based Fiber-Optic Distributed Sensor Using Dynamic Difference Attenuation Recognition

**DOI:** 10.3390/s20236922

**Published:** 2020-12-03

**Authors:** Jian Li, Xinxin Zhou, Mingjiang Zhang, Jianzhong Zhang, Lijun Qiao, Le Zhao, Zitong Yin

**Affiliations:** 1 Key Laboratory of Advanced Transducers and Intelligent Control Systems (Ministry of Education and Shanxi Province), Taiyuan University of Technology, Taiyuan 030024, China; lijian0143@link.tyut.edu.cn (J.L.); zhouxinxin0930@link.tyut.edu.cn (X.Z.); zhangjianzhong@tyut.edu.cn (J.Z.); qiaolijun@tyut.edu.cn (L.Q.); zhaole0824@link.tyut.edu.cn (L.Z.); yufuhao0939@link.tyut.edu.cn (Z.Y.); 2 College of Physics and Optoelectronics, Taiyuan University of Technology, Taiyuan 030024, China

**Keywords:** fiber sensor, Raman scattering, temperature resolution, temperature demodulation

## Abstract

There is an optical interference noise in the conventional Raman-based fiber-optics distributed sensing, which results in a poor temperature resolution performance. In addition, the traditional whole-fiber demodulation principle complicates the operation steps of the system. In this paper, a novel dynamic difference attenuation recognition (DDAR) principle is operated in the DDP scheme (dual demodulation principle) and the SDP scheme (self-demodulation principle) respectively. It not only helps to eliminate the optical interference noise, but also omits the whole-fiber calibration process. In this experiment, a temperature resolution of 0.30 °C (17.0 km) is achieved through using the DDP scheme based on the DDAR principle, and the measurement time can be shortened to 1.5 s. Meanwhile, a temperature resolution of 0.18 °C (17.0 km) is obtained for the SDP scheme under the DDAR principle. The SNR of DDP and DSP schemes can be optimized to 12.82 dB and 13.32 dB by the proposed DDAR technology. Furthermore, the temperature resolution performance under a large temperature measurement range (0–1000 °C) is theoretically analyzed. The results indicate that the temperature responsivity for DDP and SDP schemes are parabolic and linear type respectively, which causes the temperature resolution of the two schemes to show a different trend with the change of temperature. The proposed DDAR method also can improve the temperature resolution in such a large temperature measurement range.

## 1. Introduction

The Raman-based distributed fiber sensing exploits Raman-optics scattering effect along the sensing fiber to obtain the spatially distributed temperature profiles [1]. The most common Raman-based distributed temperature sensor (Raman-based DTS) employs spontaneous Raman-optics effect through measurements of the Stokes and anti-Stokes backscattered components. The Raman-based DTS for distributed temperature monitoring has been a hot research topic throughout the years [1,2,3,4,5,6,7,8]. It has been employed in a large variety of application areas due to the advantages of distributed measurement, such as fire monitoring [5], power grid [6], and gas pipeline [7,8] detection, etc.

A high-performance Raman-based DTS capable of measuring ambient temperature must be selected according to many different criteria, such as temperature accuracy [2], sensing distance [9], spatial resolution [10], measurement time [5], and temperature resolution [11], etc. The temperature resolution is known as the smallest temperature range that the sensors can resolve. It is one of the important factors in the fields of industrial temperature monitoring [7,12,13]. For example, in the pipeline leakage monitoring field, the sensors with a better temperature resolution can accurately detect the leakage position when the amount of leakage is small [7]. The sensor can calculate the cable carrying capacity and cable current by using the temperature characters in the smart grid field [12]. In order to achieve the above applications, all the temperature profiles are needed to be detected with a temperature resolution better than 1.00 °C. The current technical indicators of temperature resolution for Raman-based DTS cannot meet this requirement.

In Raman-based DTS, the intensity of the Raman backscattered component is about 60–70 dB weaker than the incident light [3] leading to a poor signal-to-noise ratio (SNR), which is the major limiting factor of the temperature resolution performance. For enhancing the temperature resolution performance, some novel demodulation schemes are proposed [11,12,13,14,15,16,17,18,19,20,21]. For example, a loop measurement scheme using Stokes and anti-Stokes components is presented [11,14]. In this scheme, the temperature distribution with 100 k time-averaged traces has been measured and then normalized in two directions with a total measurement time of 40 s. The temperature resolution with 1.50 °C and 1.10 °C are achieved in the standard and anti-Stokes trace-based traces loop configuration, respectively. However, twice the length of the sensing fiber is required in this scheme. Furthermore, increasing the spontaneous Raman scattering threshold in the sensing fiber can also optimize the temperature resolution. A few mode fiber (FMF) Raman-based DTS system is proposed [3]. The 4 mode and 2 mode FMF can respectively obtain the temperature resolution of about 7.0 °C and 6.0 °C, and these temperature signals are averaged 60 k times during 80 s. Moreover, some optical coding schemes based on either directly or externally modulated semiconductor lasers in MMFs and SMFs [15,16,17] have been proposed. The cyclic coding scheme is enabling the use of high-power pulsed-laser technology to improve the SNR. It has achieved a temperature resolution of 3 °C over a range of 26 km in 30 s of measurement [17]. In addition to the above-mentioned schemes, some novel denoising algorithms have been applied to the Raman-based DTS [1,18,19,20,21]. These denoising methods can optimize the measurement performance by improving the SNR of the Raman signal extracted from the acquisition system. The wavelet denoising algorithm can achieve a distributed temperature measurement along 7 km long range with a temperature resolution of 1.60 °C [19]. 

The above methods can effectively improve the temperature resolution performance, but its special optical mechanism makes the measurement time longer (even reaches up to 80 s), which cannot meet the requirement of real time distributed temperature monitoring. In the conventional Raman-based DTS, a Raman compensation component along the whole fiber-line is used to compensate the fiber attenuation for the extraction of the absolute temperature values in the measurement stage. This compensation component requires the entire sensing fiber to be placed in a constant temperature environment before measurement. If the sensing fiber or any device is replaced during the measurement stage, the sensor needs to be recalibrated, which makes the measurement process more complicated. Meanwhile, this Raman compensation component will bring a lot of additional optical interference noise, it will ultimately affect the temperature resolution performance. 

In this research, we propose and experimentally demonstrate a novel dynamic difference attenuation recognition (DDAR) method which can effectively eliminate the optical interference noise and optimize the temperature resolution and the SNR. In this experiment, the distributed temperature measurement and theoretical analysis based on the dual-demodulation and self-demodulation principles are carried out by using the 17.0 km graded index multimode sensing fiber. The temperature resolutions are improved in the Raman-based schemes by using the DDAR method. The whole-fiber calibration process is omitted. Moreover, the temperature resolution performances under a large temperature measurement range are theoretically analyzed. The simulation results show that the temperature resolution performances for the DDP and SDP schemes present different characteristics with the change of the measured temperature. It provides a new solution to the temperature resolution improvement for a large temperature measurement range.

## 2. Experimental Setup and Results Based on DDP Scheme with DDAR 

The Raman-based DTS employs spontaneous Raman-optics effect through detecting the Raman backscattered anti-Stokes and Stokes components [17,18,19,20,21]. There are two types of temperature demodulation principles, dual demodulation principle [22,23,24,25] (DDP), and self-demodulation principle [11,14,26] (SDP). The system based on the DDP scheme uses the intensity ratio of anti-Stokes over Stokes light for detecting the surrounding environmental temperature. The SDP scheme only uses the Raman anti-Stokes backscattered light to extract the temperature profiles. These two schemes are described in detail below.

### 2.1. The Experimental Setup and Results Based on DDP Scheme 

**Figure 1:**
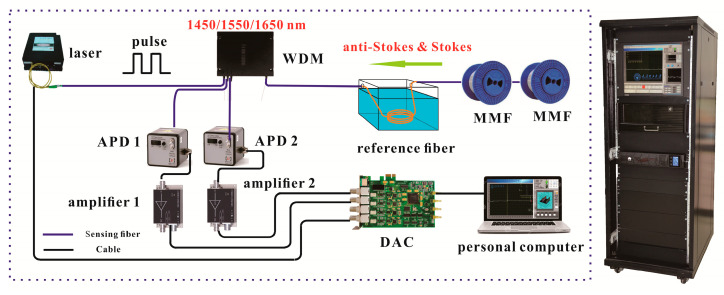
The experimental setup based on dual demodulation principle (DDP) scheme and Raman- based DTS prototype. APD: Avalanche Photodiode; WDM: Wavelength Division Multiplexing; MMF: Multimode Fiber; DAC: Data Acquisition Card.

Figure 1 displays the Raman-based DTS experimental setup based on DDP scheme. Among them, the DDP scheme includes of a pulsed laser, a Raman WDM, APD, amplifiers, DAC, personal computer, reference fiber and fiber under tests (FUTs). The device details are shown in Table 1. In Raman-based DTS, when the pulsed laser enters the sensing fiber, the Raman scattering occurs at each point along the sensing fiber. The Raman-based DTS is based on the optical time domain reflectometer (OTDR) principle to locate the temperature signal. When the temperature along the fiber increases, the intensity of the Raman scattered intensity at that point will increase. Therefore, the Raman anti-Stokes intensity is modulated by surrounding temperature. Finally, the DAC and personal computer performs the temperature demodulation by collecting the backward Raman scattered light. Moreover, a Raman-based DTS prototype based on the DDP scheme is also developed with the above experimental device.

The conventional temperature demodulation method (DDP scheme) [27] is shown in the Equation (1).

(1) $$T=\frac{1}{\left(\frac{1}{T_{c}}+\frac{1}{T_{o}}-\frac{1}{T_{co}}\right)-\frac{k}{h\Delta v}\ln\left(\frac{\phi_{sc}{}_{o}\phi_{a}{}_{o}\phi_{ac}\phi_{s}}{\phi_{aco}\phi_{so}\phi_{sc}\phi_{a}}\right)}$$

In the whole-fiber calibration stage, the *ϕ_aco_* and *ϕ_sco_* are for the anti-Stokes and Stokes lights at the reference fiber. The *ϕ_ao_* and *ϕ_so_* are the anti-Stokes and Stokes lights at the sensing fiber. *T_o_* and *T_co_* are the temperatures of the sensing fiber and reference fiber. In the measurement stage, the *ϕ_a_* and *ϕ_s_* are the lights of anti-Stokes and Stokes respectively. The *ϕ_ac_* and *ϕ_sc_* are the lights of anti-Stokes and Stokes at the reference fiber. *T_c_* is for the temperature of the reference fiber. The *h* is the Planck’s constant, Δv is the Raman frequency shift, *k* is the Boltzmann constant, *T* is the absolute temperature.

**Figure 2:**
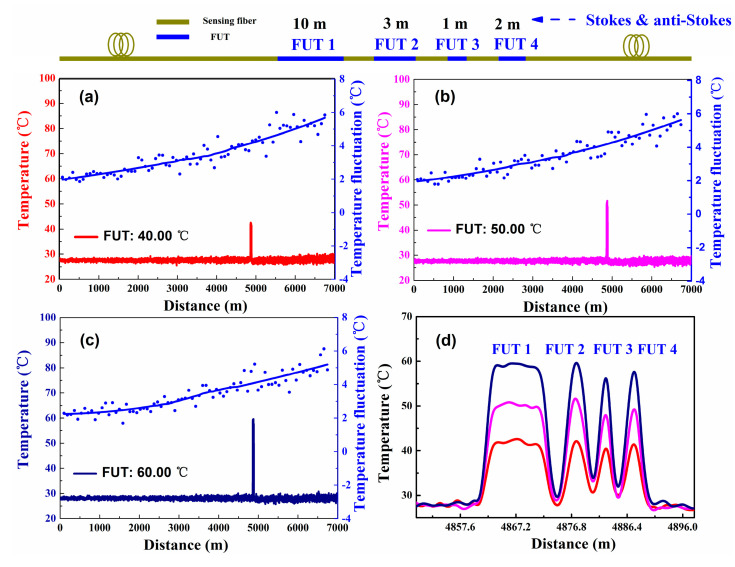
The distributed temperature and temperature resolution measurement for the DDP scheme, which use the conventional demodulation method under the (**a**) 40.00 °C, (**b**) 50.00 °C, (**c**) 60.00 °C. (**d**) The temperature components in the proximity of the fiber under tests (FUTs).

The distributed temperature experiment based on DDP scheme is performed. Among them, the sensing fiber consists of four sections (FUT 1, FUT 2, FUT 3, and FUT 4). The temperatures of FUTs are all set at 40.00 °C, 50.00 °C, and 60.00 °C by the TCC. In addition, the rest of the sensing fiber is placed at room environment (the room temperature is about 28.00 °C). Then the temperature information along the 7.0 km fiber are detected according to Equation (1). The measurement results are shown in Figure 2a–c. The Figure 2d shows an enlarged part of the temperature measurement result. In this experiment, the temperature resolution distributions are obtained by calculating the temperature fluctuation range within a window of 50 m or the standard deviation of the measured temperature [3,11]. As shown in the Figure 2a–c, the blue spot curves display for the temperature resolution along the 7 km sensing fiber (by calculating the temperature fluctuation range) with the temperature results averaged at 10 k times. Because of the SNR deteriorates with the increase of the sensing distance, the measurement result exhibits a worse temperature resolution at the end of fiber compared to the start position of the fiber. The experimental results show that the temperature resolution is 2.45 °C and 6.00 °C under a sensing distance of 1.0 km and 7.0 km, this lower temperature resolution performance limits the application of Raman-based DTS. 

### 2.2. Temperature Demodulation Principle and Temperature Resolution Analysis 

**Figure 3:**
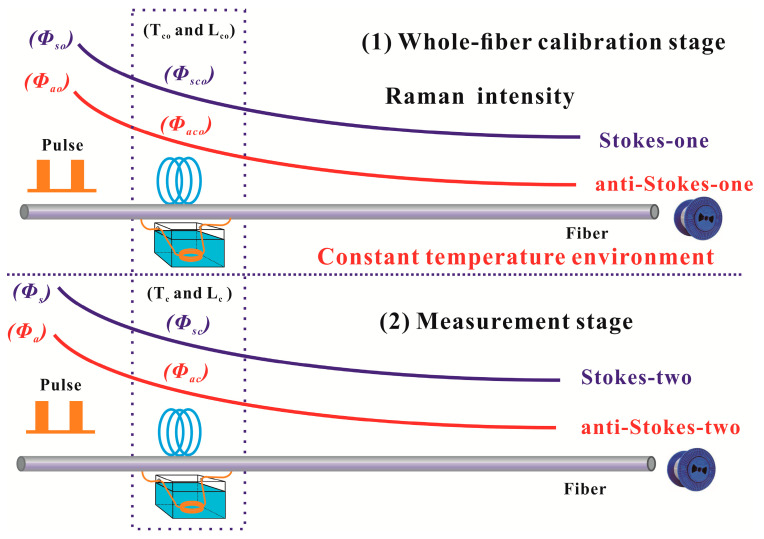
The traditional whole-fiber demodulation schematic diagram for DDP scheme.

In the conventional demodulation method, the Raman-based DTS system needs two sections of Raman intensity signal for temperature extraction. The conventional demodulation schematic diagram is shown in the Figure 3. Among them, the part of the Raman intensity comes from the measurement stage, and the other part comes from the whole-fiber calibration stage before the measurement. This conventional demodulation mechanism requires the system to place all the sensing fibers under a constant temperature condition for calibration before measurement. Most importantly, the extracted Raman signal in the whole-fiber calibration stage is about 30 dB smaller than the Rayleigh scattering signal. The SNR of the Raman scattering signal collected by the DAC in the whole-fiber calibration stage is weak. Among them, a large of noise are doped into the Stokes-one and anti-Stokes-one channels at the whole-fiber calibration stage. Thence, when the conventional demodulation method uses this method with weak SNR to extract the temperature information, the system will inevitably cause a low temperature resolution performance. As shown in the Equation (1), in the conventional DDP scheme, two sections of the intensity ratio of Raman demodulation signal (anti-Stokes over Stokes light, *ϕ_a_/ϕ_s_* and *ϕ_ao_/ϕ_so_*) are used to extract the temperature components along the sensing fiber [27,28]. The *ϕ_ao_/ϕ_so_* (Stokes-one and anti-Stokes-one traces) is used to compensate the fiber attenuation of the whole fiber-line. If the sensing fiber or any device is replaced during the measurement stage, the system must need to be re-calibrated under the constant temperature environment. It makes the operation steps of Raman-based DTS more complicated. The existing Raman-based DTS has problems of poor resolution performance at low temperatures and complicated measurement steps.

### 2.3. The Novel DDAR Demodulation Principle for DDP Scheme 

**Figure 4:**
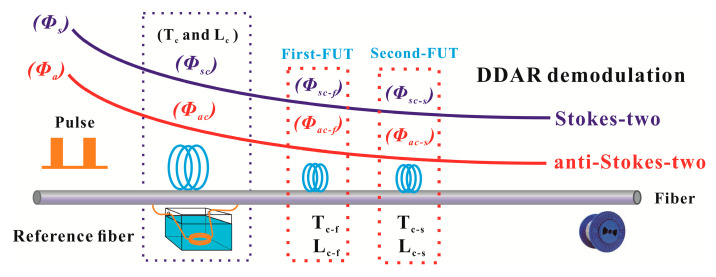
The dynamic difference attenuation recognition (DDAR) demodulation principle for DDP scheme.

In order to avoid the additional optical interference noise and omit the whole-fiber calibration process, we propose a novel dynamic difference attenuation recognition (DDAR) method. We only need one section of the intensity ratio (*ϕ_a_/ϕ_s_*, Stokes-two and anti-Stokes-two) to perform the temperature demodulation. The novel DDAR-based temperature demodulation method includes two parts, the attenuation recognizes stage and the measurement stage. These two steps can be performed simultaneously, as shown in the Figure 4. In the attenuation recognition stage, the intensity ratio of the First-FUT and Second-FUT (the same type of fiber) are used to calculate the fiber attenuation. The intensity ratio of the first section (anti-Stokes over Stokes, First-FUT) is referred as follows:

(2) $$\frac{\phi_{a\mathrm{c-}f}}{\phi_{s\mathrm{c-}f}}=\frac{K_{a}}{K_{s}}{\left(\frac{\upsilon_{a}}{\upsilon_{s}}\right)}^{4}\exp\left(-\frac{h\Delta v}{kT_{\mathrm{c-}f}}\right)\exp\left[\int_{0}^{L_{\mathrm{c-}f}}\left(\alpha_{s}(L)-\alpha_{a}(L)\right)dL\right]$$

The intensity ratio of the second section (Second-FUT) is referred as follows:

(3) $$\frac{\phi_{a\mathrm{c-}s}}{\phi_{s\mathrm{c-}s}}=\frac{K_{a}}{K_{s}}{\left(\frac{\upsilon_{a}}{\upsilon_{s}}\right)}^{4}\exp\left(-\frac{h\Delta v}{kT_{\mathrm{c-}s}}\right)\exp\left[\int_{0}^{L_{\mathrm{c-}s}}\left(\alpha_{s}(L)-\alpha_{a}(L)\right)dL\right]$$

where the *T_c-f_* and *T_c-s_* display for the temperature of the First-FUT and Second-FUT. The *L_c-f_* and *L_c-s_* display for the distance of the First-FUT and Second-FUT. Then the fiber attenuation coefficient can be calculated according to the Equations (2) and (3).

(4) $$\alpha_{DDP}=\alpha_{s}-\alpha_{a}=\frac{\ln\left(\frac{\phi_{s\mathrm{c-}s}}{\phi_{a\mathrm{c-}s}}\frac{\phi_{s\mathrm{c-}f}}{\phi_{a\mathrm{c-}f}}\right)}{\left(L_{\mathrm{c-}s}-L_{\mathrm{c-}f}\right)}+\frac{\ln\left[\frac{1-\exp(-h\Delta v/kT_{\mathrm{c-}s})}{1-\exp(-h\Delta v/kT_{\mathrm{c-}f})}\right]-\ln\left[\frac{\exp(h\Delta v/kT_{\mathrm{c-}s})-1}{\exp(h\Delta v/kT_{\mathrm{c-}f})-1}\right]}{L_{\mathrm{c-}s}-L_{\mathrm{c-}f}}$$

During the measurement stage, the intensity ratio of the Raman along the sensing fiber and the reference fiber can be defined as

(5) $$\frac{\phi_{a}}{\phi_{s}}\frac{\phi_{sc}}{\phi_{ac}}=\exp\left[-\frac{h\Delta v}{k}\left(\frac{1}{T}-\frac{1}{T_{c}}\right)\right]\exp\left[\int_{L_{c}}^{L}\left(\alpha_{s}(L)-\alpha_{a}(L)\right)dL\right]$$

Then the temperature components along the fiber-line can be calculated by using the Equations (4) and (5), as shown in the Equation (6).

(6) $$\frac{1}{T}=\left[\ln\left(\frac{\phi_{a}\phi_{sc}}{\phi_{s}\phi_{ac}}\right)-\alpha_{\mathrm{DDP}}\left(L_{c}-L\right)\right]/\left(-\frac{h\Delta v}{k}\right)+\frac{1}{Tc}$$

The proposed method uses the reference temperature signals generated by First-FUT and Second-FUT to demodulate the distributed attenuation information of the sensing fiber. Both the First-FUT and Second-FUT are all included in the measurement fiber-line. Then the attenuation information of the sensing fiber is introduced into Raman scattering signal to extract distributed temperature information. The advantage of this method is that it does not require the additional compensation components along the whole fiber-line, which can omit the whole-fiber calibration process. Moreover, the proposed method can be compatible with many types of optical fibers, such as MMF and single mode fibers (SMF). Due to the whole-fiber calibration stage is omitted, the system is not affected by the replacement of sensing fiber and device, which can be effectively used for a long time.

### 2.4. The Temperature Resolution Results for DDP Scheme Using the DDAR 

**Figure 5:**
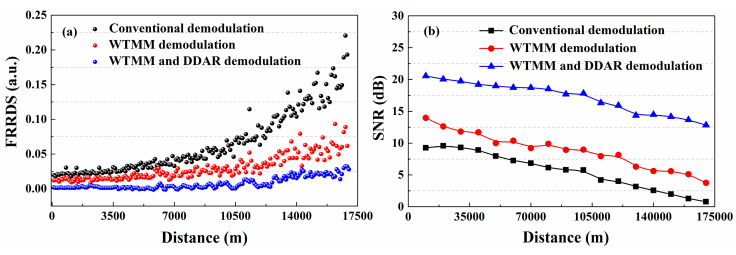
The (**a**) fluctuation range of Raman demodulation signal (FRRDS) trends and (**b**) signal-to-noise ratio (SNR) measurement results for DDP scheme using the different demodulation methods.

In the Raman-based DTS, the fluctuation range of Raman demodulation signal (FRRDS) can be represented by the SNR. The experimental results proved that the FRRDS for the conventional demodulation method is 0.22 at 17.0 km as shown in the black dotted curve in Figure 5a, while the SNR is 0.79 dB. The wavelet transform modulus maxima (WTMM) is an effective denoising method for Raman-based DTS. After the denoising of the WTMM, the FRRDS is 0.08 at 17.0 km, as shown in the red dotted curve in Figure 5a, and its SNR is 3.75 dB. The DDAR demodulation method can avoid the additional Raman noise-optics interference. As shown by the blue dotted curve in Figure 5a, the FRRDS can optimize to 0.031 at 17.00 km by using the DDAR method combined with the WTMM. Compared with the conventional demodulation method, the SNR for DDAR demodulation method is improved to 12.82 dB, as shown in Figure 5b. Experimental results show that the SNR of the collected Raman demodulated signal can be effectively improved for DDP scheme.

**Figure 6:**
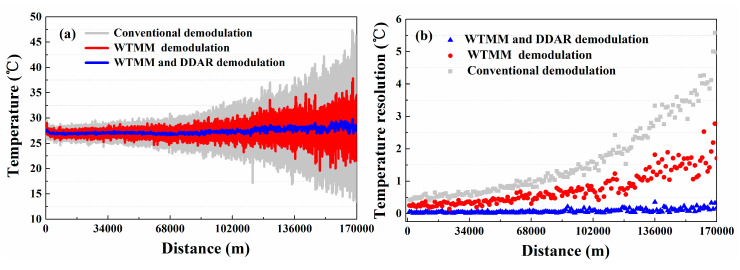
The (**a**) temperature measurement results and (**b**) the temperature resolution using the conventional demodulation, wavelet transform modulus maxima (WTMM), and DDAR demodulation methods for DDP scheme.

**Figure 7:**
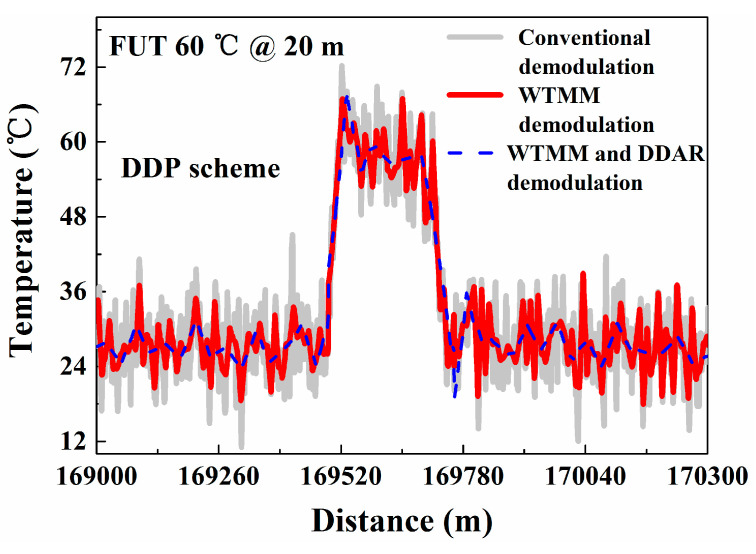
Temperature measurement results of FUT.

To make a sensible performance comparison between the conventional and the proposed methods, another contrast experiment is conducted. In this experiment, we compare the temperature resolution (standard deviations of temperature) based on the conventional demodulation method and the proposed method. The FUTs are placed in a TCC which keeps the temperature at 27.0 °C. After running the Raman-based DTS system stably, the distributed temperature is measured using the conventional demodulation method based on Equation (1) and the proposed demodulation method based on Equation (6), respectively. The measured temperature trends and temperature resolution along the whole 17.0 km sensing fiber are shown in Figure 6a,b. The temperature components are averaged 10 k times. The grey line and red line represent the temperature measured using the conventional demodulation method and WTMM-based demodulation method, respectively. The blue line shows the temperature components demodulated by combining the DDAR method and WTMM method. The temperature resolution is optimized from 0.61 °C to 0.05 °C at 1.0 km. The temperature resolution with 5.57 °C is optimized to 0.30 °C at 17.0 km. In addition, we place a FUT (20 m) with a position of 17.0 km into a high-precision constant temperature bath (Talent, BH8001, its temperature control range is 20.0–60.0 °C). Then the temperature of FUT is set to 60 °C in the experiment. The experimental results also show that the temperature fluctuation range of proposed method is better than conventional demodulation method and WTMM demodulation method, as shown in the Figure 7. 

Note that our proposed method provides an enhanced temperature resolution compared with conventional configuration. The most important thing is that the proposed method can improve the SNR without deteriorating the measurement time of the system, and omit the whole-fiber calibration process, which keeps the measurement time at 1.5 s under the premise of the obtained temperature resolution. Compared with the methods described in the introduction, the measurement time of the proposed method has an obvious advantage. Furthermore, the proposed DDAR method cannot solve the problem of the additional fiber losses that affects the temperature measurement accuracy. Therefore, in the practical applications, it is necessary to locate these positions where the temperature measurement error is caused by factors such as fiber bending and fusion before the distributed temperature measurement.

## 3. Experimental Setup and Results Based on SDP Scheme with DDAR 

### 3.1. The Experimental Setup and Results Based on SDP Scheme

**Figure 8:**
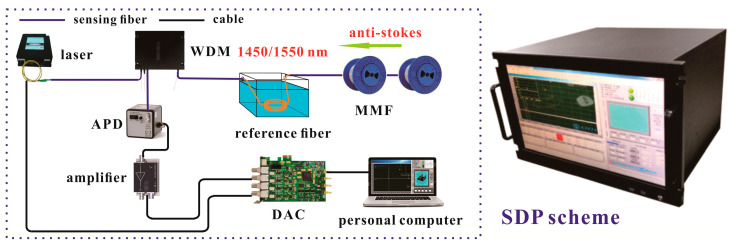
The experimental setup for SDP scheme. APD: Avalanche Photodiode; Amp: Amplifier; WDM: Wavelength Division Multiplexing; MMF: Multimode Fiber; DAC: High-speed Data.

The experimental setup for the SDP scheme is shown in Figure 8. Compared to the collection system with DDP scheme, the SDP scheme only includes one APD and an amplifier for extracting the anti-Stokes Raman component (1450 nm). It means that the Raman WDM only needs to filter out the anti-Stokes light for demodulating the temperature information. The remaining devices are basically consistent with the DDP scheme. The conventional temperature demodulation algorithm for SDP scheme [19] is shown in the Equation (7).

(7) T=ln{[exp(hΔνkTo)−1][exp(hΔνkTc)−1][exp(hΔνkTco)−1](ϕaϕacoϕaoϕac)+1}−1(hΔνk)

**Figure 9:**
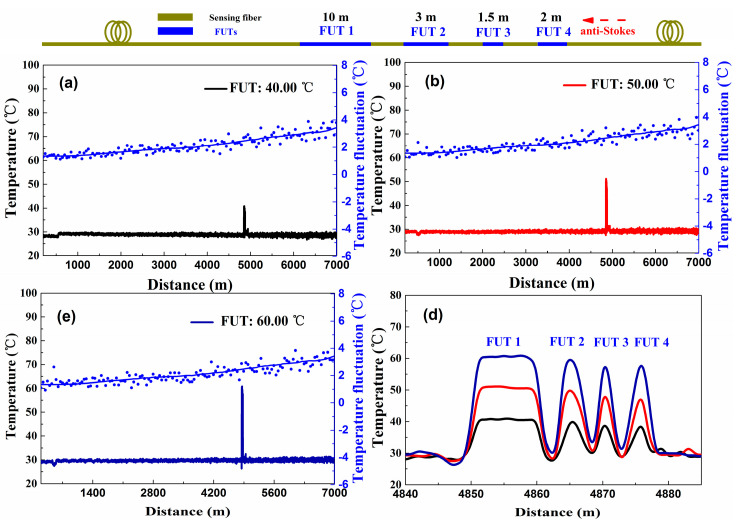
The distributed temperature and temperature resolution measurement for SDP scheme, using the conventional demodulation method under the (**a**) 40.00 °C, (**b**) 50.00 °C, (**c**) 60.00 °C. (**d**) The temperature profiles at the FUTs.

In the experiment of SDP scheme, the distributed temperature along the 7.0 km MMF is detected by using conventional demodulation method. The temperature of FUTs are set at 40.00 °C, 50.00 °C and 60.00 °C respectively by the TCC and the rest of the sensing fiber is maintained at room temperature. The temperature measurement results of overall distribution are shown in Figure 9. Figure 9a–c displays the overall distribution of the temperature measurement results. The temperature components along the 7.0 km sensing fiber are then calculated according to Equation (7). Figure 9d reports the temperature profile in the proximity of the FUTs. In addition, as shown in Figure 9a–c, the blue spot curve displays for the temperature resolution (by calculating the temperature fluctuation range) for SDP scheme along the 7 km fiber. The experimental results show that the temperature resolutions for SDP scheme are 1.27 °C and 3.80 °C under a sensing distance of 1.0 km and 7.0 km. 

### 3.2. The Novel DDAR Principle for SDP Scheme 

**Figure 10:**
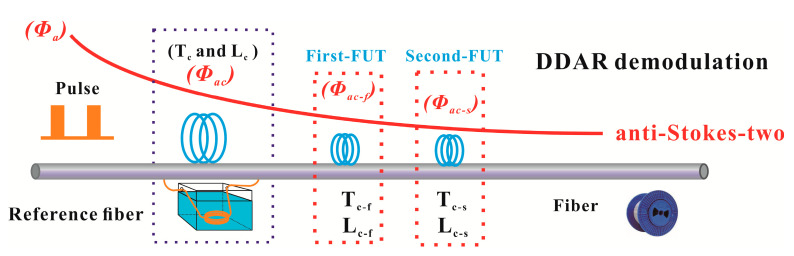
The DDAR demodulation schematic diagram for SDP scheme.

In the conventional SDP scheme, only two anti-Stokes intensities signals (*ϕ_a_* and *ϕ_ao_*, anti-Stokes-one and anti-Stokes-two) are used to extract the distributed temperature information. Compared with the operation steps of the DDP scheme, the SDP scheme also requires a whole fiber calibration process. In the whole-fiber calibration stage, the *ϕ_ao_* is applied as a Raman compensation component for calibrating the attenuation along the fiber-line [19]. Based on the analysis above, the whole-fiber calibration stage will bring a lot of additional optical interference noise in the anti-Stokes-two channel. Thus, in order to eliminate the above optical interference noise in the anti-Stokes-two channel, we also propose a DDAR-demodulation method for SDP scheme. Only one section of the anti-Stokes intensity (*ϕ_a_*, anti-Stokes-two) is used to perform the temperature demodulation, as shown in Figure 10.

The DDAR-based demodulation method for SDP scheme also includes two parts, the attenuation recognizes stage and the measurement stage. In the attenuation recognize stage, the anti-Stoke intensity of the first section (First-FUT) is referred to as follows:

(8) $$\phi_{a\mathrm{c-}f}=K_{a}\upsilon_{a}{}^{4}{\left[\exp\left(\frac{h\Delta v}{kT_{\mathrm{c-}f}}\right)-1\right]}^{-1}\exp\left[\int_{0}^{L_{\mathrm{c-}f}}-\left(\alpha_{o}(L)+\alpha_{a}(L)\right)dL\right]$$

The intensity of the second section (Second-FUT) is referred as follows:

(9) $$\phi_{a\mathrm{c-}s}=K_{a}\upsilon_{a}{}^{4}{\left[\exp\left(\frac{h\Delta v}{kT_{\mathrm{c-}s}}\right)-1\right]}^{-1}\exp\left[\int_{0}^{L_{\mathrm{c-}s}}-\left(\alpha_{o}(L)+\alpha_{a}(L)\right)dL\right]$$

Then the attenuation coefficient of the sensing fiber is calculated according to the Equations (8) and (9).

(10) $$\alpha_{SDP}=\alpha_{o}+\alpha_{a}=-\ln\left[\frac{\phi_{a\mathrm{c-}f}}{\phi_{a\mathrm{c-}s}}\frac{\exp\left(h\Delta v/kT_{\mathrm{c-}f}\right)-1}{\exp\left(h\Delta v/kT_{\mathrm{c-}s}\right)-1}\right]{\left(L_{\mathrm{c-}f}-L_{\mathrm{c-}s}\right)}^{-1}$$

During the measurement stage, the anti-Stokes intensity ratio along the sensing fiber and reference fiber can be defined as

(11) $$\frac{\phi_{a}}{\phi_{ac}}=\frac{\exp\left(h\Delta v/kT_{c}\right)-1}{\exp\left(h\Delta v/kT\right)-1}\exp\left[\int_{L_{c}}^{L}-\left(\alpha_{o}(L)+\alpha_{a}(L)\right)dL\right]$$

Then the temperature information along the fiber-line is demodulated by using Equations (10) and (11), as shown in Equation (12). Both First-FUT and Second-FUT are included in the measurement fiber-line. In addition, due to the omitted whole-fiber calibration stage, SDP scheme is not affected by sensor fiber and device replacement, so it can be used effectively for a long time.

(12) $$\frac{1}{T}=\langle \ln\left\{\left[\exp\left(\frac{h\Delta v}{kT_{c}}\right)-1\right]\exp\left[\int_{L_{c}}^{L}-\left(\alpha_{o}(L)+\alpha_{a}(L)\right)dL\right]\frac{\phi_{a}}{\phi_{ac}}\right\}+1\rangle \frac{k}{h\Delta v}$$

### 3.3. The Temperature Resolution for SDP Scheme Using the DDAR 

**Figure 11:**
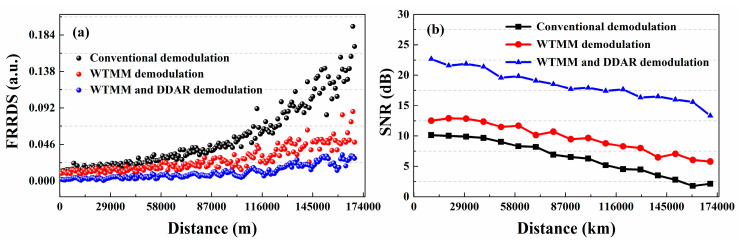
(**a**) The FRRDS trends along the sensing distance for SDP scheme. (**b**) The SNR measurement results using the different methods.

In the Raman-based DTS with SDP scheme, as shown in the black dotted curve in Figure 11a, the FRRDS based on the Equation (7) is 0.194 at 17.0 km, and its SNR is 2.13 dB. After the denoising of the WTMM, the FRRDS is 0.087 at 17.0 km, while the SNR is 5.80 dB. However, the FRRDS can improve to 0.004 by using the DDAR method combined with the WTMM, as shown in the red dotted curve in Figure 11a. Compared with the conventional SDP temperature demodulation method, its SNR can be optimized to 13.32 dB. Experimental results show that the SNR of the collected Raman demodulated signal can be effectively improved for SDP scheme.

**Figure 12:**
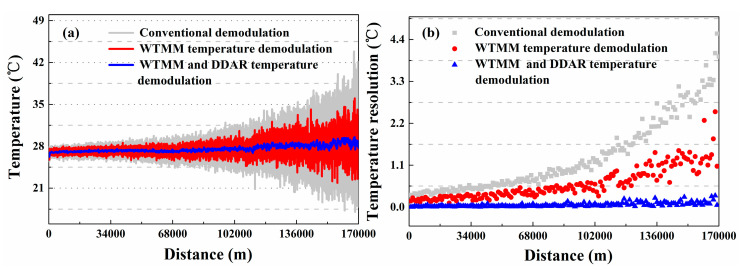
The (**a**) distributed temperature trends and (**b**) temperature resolutions for SDP scheme using the conventional, WTMM and DDAR demodulation methods.

**Figure 13:**
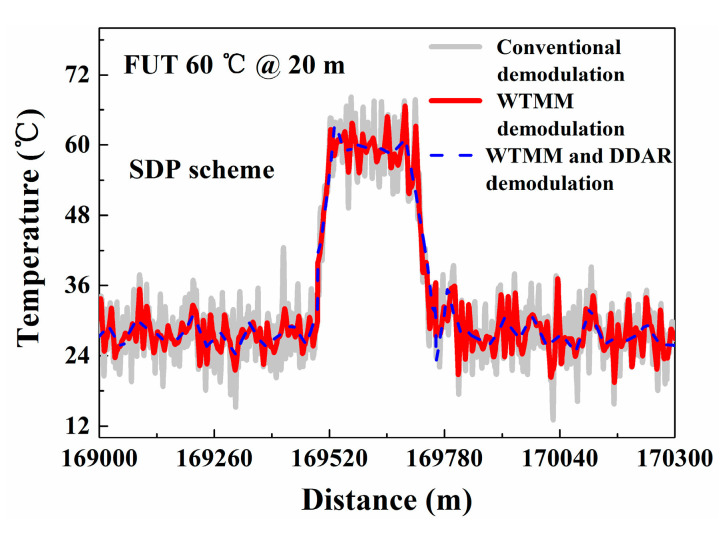
Temperature measurement results of FUT.

In the temperature measurement experiment, we also compare the conventional demodulation method and the proposed method as their temperature resolution and standard deviations for verifying the improvement of temperature resolution. The temperature is measured by using a conventional demodulation method based on Equation (7) and the proposed demodulation method based on Equation (12), respectively. The distributed temperature trends and the temperature resolution along the sensing fiber using different methods are shown in Figure 12a,b. The temperature resolution is optimized from 0.40 °C to 0.04 °C at 1.0 km and from 4.55 °C to 0.18 °C at 17.0 km. Experiment results show that the measurement temperature resolution of Raman-based DTS for SDP scheme also has been optimized. In addition, its measurement time is still 1.5 s. Furthermore, in SDP scheme we also place the FUT (20 m) with a location of 17.0 km in a high-precision constant temperature bath, and set its temperature is 60.0 °C. The results show that the temperature fluctuation range of proposed method is better than conventional demodulation method and WTMM demodulation method, as shown in the Figure 13.

## 4. Simulation of Temperature Resolution Performance under a Large Temperature Measurement Range

### 4.1. Theoretical Analysis of Temperature Resolution Performance for DDP Scheme

The Raman-based DTS performance also includes the largest temperature measurement range, especially for the needs of fire detection [29,30]. The characteristics of the temperature resolution under a large temperature measurement range is also extremely important, which has not been studied in previous work. In Raman-based distributed fiber sensing, the temperature measurement range of the system depends on the characteristics of the sensing fiber. The ordinary sensing fiber can only evaluate the measurement temperature range of −20 °C to 120 °C. Some special sensing fibers can be used for high temperature or cryogenic temperature measurement. For example, The Polyimide coated optical fiber can achieve a high temperature measurement with 350 °C. Moreover, there is a special type of sensing fiber based on featuring metallic coating for high temperature operation. Marianne S. Peixoto e Silva et al. presents the experimental evaluation of a Raman-based DTS over a wide temperature range, from −196 °C to 400 °C [31]. This is the first evaluation of these aspects on Raman-based DTS in such a wide temperature range, much wider than in previous publications. Practically, the silica-based optical fiber material degrades right above 1000 °C. Therefore, we limit the upper limit of the simulated experiment temperature to 1000 °C. In this paper, we use the simulation experiments for distributed detection of high temperature.

In this section, the theoretical analysis of temperature resolution performance for DDP scheme is analyzed. Due to the limitations of experimental conditions, we theoretically studied the relationship between Raman demodulation signal (*A_DDP_*) and measurement temperature (*T*). The high-temperature measurement simulation is measured by LabVIEW and MATLAB software. The Equation (13) represents the function of *A_DDP_* after the operational temperature demodulation.

(13) $$A_{DDP}=\frac{\phi_{ac}\phi_{s}}{\phi_{sc}\phi_{a}}=\exp\left\{\left[\left(\frac{1}{T_{c}}-\frac{1}{T}\right)\frac{h\Delta v}{k}\right]+\alpha_{DDP}\left(L_{c}-L\right)\right\}$$

where the *T_c_* represents the temperature value of the reference fiber at the measurement stage and be used for calibrating the influence of the APD gain fluctuation on the temperature demodulation results. The remaining parameters are known constants at the measurement stage. Thence, the *A_DDP_* is totally depending on the measurement temperature. 

**Figure 14:**
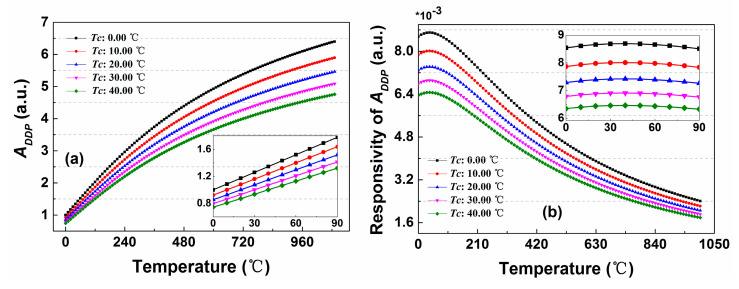
(**a**) The relationship of *A_DDP_* and the measurement temperature under the different calibration temperatures. (**b**) The responsivity of *A_DDP_* to the measurement temperature.

Based on the above analysis, some numerical simulation experiments are performed. Firstly, the relationship between *A_DDP_* and measurement temperature is studied under different calibration temperatures (*T_C_*), as shown in the Figure 14a. It can be concluded that the *A_DDP_* shows an increasing trend as the measured temperature increases, which is the essence of Raman temperature effect. Most importantly, the increasing rate of *A_DDP_* will gradually slow down along with the growth of measurement temperature, which means that the temperature responsivity of *A_DDP_* reveals a gradually decreasing trend with the increase of measurement temperature. Then a simulation analysis of the responsivity of *A_DDP_* to the measurement temperature is studied according the Equation (14).

(14) $$\frac{d\left(A_{DDP}\right)}{d\left(T\right)}=\exp\left\{\left[\left(\frac{1}{T_{c}}-\frac{1}{T}\right)\frac{h\Delta v}{k}+\alpha_{DDP}\left(L_{c}-L\right)\right]\right\}*\frac{h\Delta v}{kT^{2}}$$

As shown in the Figure 14b, it can be observed that the responsivity of *A_DDP_* to the measured temperature is parabolic, and reaches its peak at 40.00 °C. Then it maintains a downward trend in the temperature range of 40.00–1000.00 °C, which means that the temperature measurement performance of system, such as temperature resolution will become worse as the measured temperature increases. It can be interpreted in a way that with the increase of measurement temperature, the Raman temperature effect (temperature response) gradually decreases, but the noise of the system does not change. 

**Figure 15:**
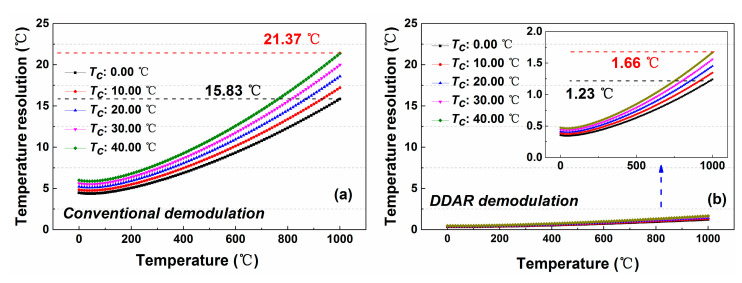
The simulation results of the temperature resolution for DDP scheme by using the (**a**) conventional and (**b**) DDAR demodulation methods.

Figure 15a displays for the trend of temperature resolution with the measured temperature. In this experiment, the noise coefficient for DDP scheme with the conventional demodulation method is 0.038, which is substituted into the temperature resolution calculation in the Figure 13. The experimental results indicate that the temperature resolution will deteriorate as the measured temperature increases, and it decreases to 21.37 °C when the temperature rises to 1000.00 °C. The DDAR demodulation method will also maintain the same characteristics, as shown in the Figure 15b because the temperature demodulation function for these two methods are basically the same. However, due to the improved SNR (The noise coefficient for DDP scheme with DDAR demodulation method is 0.006 at 8.0 km.), its temperature resolution can be improved to 1.66 °C under the same measurement conditions.

In addition, the difference of the reference temperature also affects the temperature resolution performance. As shown in the Figure 15b, The responsivity of *A_DDP_* to the measured temperature will increase when the reference temperature is kept at a lower value, which leads to the temperature resolution performance becoming better, as shown in the Figure 13. Therefore, in the actual temperature monitoring, the measurement performance of the system can be improved by lowering the reference temperature value.

### 4.2. Theoretical Analysis of Temperature Resolution Performance for SDP Scheme 

In this section, we also theoretically study the relationship between Raman demodulation signal (*A_SDP_*) and measurement temperature (*T*) for SDP scheme. The Equation (15) represents the function of *A_SDP_* after the operational temperature demodulation.

(15) $$A_{SDP}=\frac{\phi_{a}}{\phi_{ac}}=\frac{\exp\left(h\Delta v/kT_{c}\right)-1}{\exp\left(h\Delta v/kT\right)-1}\exp\left[\int_{L_{c}}^{L}-\alpha_{SDP}dL\right]$$

**Figure 16:**
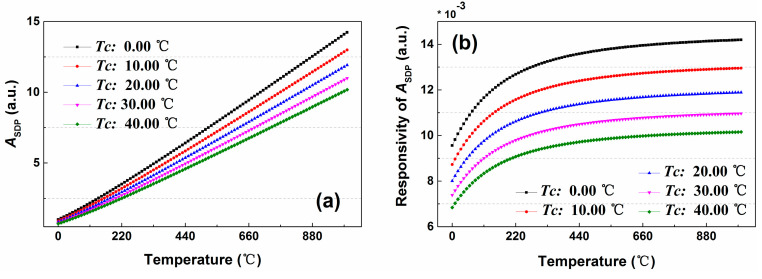
(**a**) The relationship of *A_SDP_* and the measurement temperature under the different calibration temperatures. (**b**) The responsivity of *A_DDP_* to the measurement temperature.

From the Equation (14), it can be observed that the *A_SDP_* is also entirely depending on the measurement temperature (*T*) at the measurement stage. Then the relationship between the *A_SDP_* and the measurement temperature at the different calibration temperatures (*T_c_*) is studied, as shown in the Figure 16a. Compared with the DDP scheme, the *A_SDP_* also reveals an increasing trend as the measured temperature increases. Then we analyzed the responsivity of *A_SDP_* to measurement temperature based on Equation (16).

(16) $$\frac{d\left(A_{SDP}\right)}{d\left(T\right)}=\left[\exp\left(\frac{h\Delta v}{kT_{c}}\right)-1\right]\exp\left[\int_{L_{c}}^{L}-\alpha_{SDP}dL\right]{\left[\exp\left(\frac{h\Delta v}{kT}\right)-1\right]}^{-2}\frac{h\Delta v}{kT^{2}}$$

However, the difference is that the temperature responsivity of *A_DDP_* shows an increasing trend with the growth of measurement temperature, as shown in the Figure 16b. This means the temperature resolution shows a better performance while the sensing fiber stay in a high temperature condition. The reason for this phenomenon is that the temperature modulation functions of the SDP and DDP schemes are different. 

**Figure 17:**
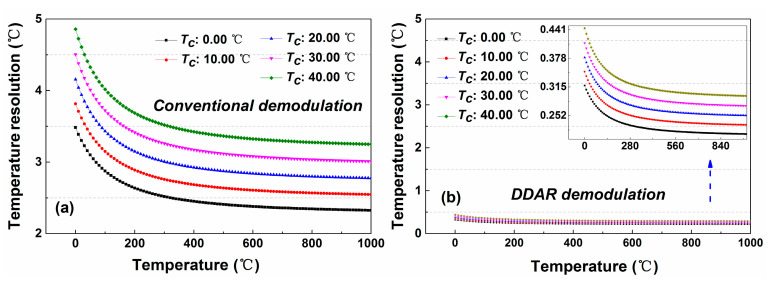
The simulation results of the temperature resolution for SDP scheme by using the (**a**) conventional and (**b**) DDAR demodulation method.

Figure 17 shows the simulation results of the temperature resolution changing with the increase of the measured temperature. The experimental results show that in the measurement range of 0–300.00 °C, the temperature resolution of the SDP scheme decreases more obviously. When the measurement temperature is higher than 300.00 °C, the downward trend becomes gentler, and finally the temperature resolution stabilizes at 2.32 °C (*T_c_* is room temperature, and the noise coefficient for SDP scheme with conventional demodulation methods is 0.033.). Through the DDAR demodulation method, the temperature resolution of the SDP scheme can be maintained at 0.21 °C under the same measurement condition. (The noise coefficient for SDP scheme with DDAR demodulation methods is 0.004.) The reduction of the reference temperature can also improve the temperature responsivity and ultimately optimize the temperature resolution performance.

## 5. Discussion

In the traditional temperature demodulation process of Raman-based DTS system, the fiber attenuation in the measurement stage and the calibration stage is consistent at a room temperature condition. However, the fiber attenuation will change under an extreme temperature condition, which causes the fiber attenuation in the calibration stage and the measurement stage to be inconsistent. This phenomenon will affect the measurement error of the system. The theoretical analysis of measurement for extreme temperature is as follows:

During the calibration stage, the Raman intensity ratio along the sensing fiber can be defined as

(17) $$\frac{\phi_{ao}}{\phi_{so}}=\frac{K_{a}}{K_{s}}{\left(\frac{\upsilon_{a}}{\upsilon_{s}}\right)}^{4}\exp\left(-\frac{h\Delta v}{kT_{o}}\right)\exp\left[\int_{0}^{L}\left(\alpha_{s}(L)-\alpha_{a}(L)\right)dL\right]$$

During the measurement stage, the fiber attenuation will change slightly due to high temperature conditions. The Raman intensity ratio along the sensing fiber at the measurement stage can be defined as

(18) $$\frac{\phi_{a}}{\phi_{s}}=\frac{K_{a}}{K_{s}}{\left(\frac{\upsilon_{a}}{\upsilon_{s}}\right)}^{4}\exp\left(-\frac{h\Delta v}{kT}\right)\exp\left[\int_{0}^{L}a_{H}\left(T\right)\left(\alpha_{s}(L)-\alpha_{a}(L)\right)dL\right]$$

The $a_{H}\left(T\right)$ is the fiber attenuation modulation factor related to temperature. The temperature expression function along the sensing fiber can be calculated by equations (17) and (18), as shown in Equation (19).

(19) $$\frac{1}{T}=\ln\left(\frac{\phi_{a}\phi_{so}}{\phi_{s}\phi_{ao}}*\frac{\exp\left[\int_{0}^{L}\left(\alpha_{s}(L)-\alpha_{a}(L)\right)dL\right]}{\exp\left[\int_{0}^{L}\alpha_{H}\left(\alpha_{s}(L)-\alpha_{a}(L)\right)dL\right]}\right)\left(-\frac{k}{h\Delta v}\right)+\frac{1}{T_{o}}$$

It can be seen from Equation (19) that the temperature information is not only modulated by the Raman signal, but also related to the fiber attenuation modulation factor. This phenomenon will affect the sensing performance of the system, including temperature resolution. Therefore, when applying the Raman-based DTS be applied to the extreme temperature measurement, the fiber attenuation should be considered in the demodulation process.

The DDAR method proposed in this paper is based on the SNR improvement to optimize the temperature resolution performance. This method cannot solve the problem of fiber attenuation changes caused by extreme temperature environments. In addition, the fiber attenuation does not show a linear relationship with the temperature change, it cannot apply the fiber attenuation modulation factor to this simulation model at the extreme temperature condition. Therefore, in order to avoid the above-mentioned problems, some special sensing fibers should be used as sensing fibers for temperature measurement. These special fibers can keep its fiber materials basically unchanged under extreme temperature conditions. Hence the boundary conditions of this simulation model are based on the constant attenuation of the fiber.

## 6. Conclusions

In this work, the distributed temperature measurement and theory analysis using the DDP and SDP schemes are experimental demonstrated. In the conventional Raman-based DTS, there is an optical interference noise due to the whole-fiber demodulation mechanism. Eventually it leads to a poor temperature resolution performance and complicates operation stage. Based on this, a novel DDAR demodulation principle is proposed. In the experiment, the temperature resolution with 0.05 °C (1.0 km) and 0.30 °C (17.0 km) are achieved in DDP scheme by using the DDAR principle. Meanwhile, the temperature resolution with 0.04 °C (1.0 km) and 0.18 °C (17.0 km) are obtained for SDP scheme under the DDAR principle. The SNR are improved to 12.82 dB (DDP) and 13.32 dB (SDP), respectively. Such a DDAR-based principle allows for the cancellation of additional optical interference noise, and the whole-fiber calibration process is also omitted. To the best of our knowledge, it means that the Raman-based DTS can ensure the best temperature resolution in such a short measurement time. 

Moreover, the temperature resolution performances under a wide temperature measurement range are theoretically analyzed by using a numerical simulation model based on the Raman-intensity responsivity to the temperature. The results indicate that the temperature responsivity for DDP and SDP schemes present a parabolic and linear increasing trend, respectively, which causes that the temperature resolution performance of these two schemes presents the different trends as the measured temperature changes. Meanwhile, the DDAR method can also be applied to optimize the temperature resolution performance in a large temperature measurement range. The research in this work provides a promising solution to the performance improvement of temperature resolution for Raman-based DTS. 

## Figures and Tables

**Table 1:** Parameters of device in the experiment.

Device	Manufacturer	Parameters
Laser	Connect Laser,	wavelength: 1550 nm
Raman filter	Xufeng Photoelectric	1550 nm/1450 nm/1650 nm
APD	FBY Photoelectric	bandwidth: 100 MHz
Amplifiers	REBES	bandwidth: 100 MHz
DAC	Jemetech	bandwidth: 100 MHz
